# Experiences of Using the Digital Support Tool MeeToo: Mixed Methods Study

**DOI:** 10.2196/37424

**Published:** 2022-10-20

**Authors:** Giulia Gaia Ravaccia, Sophie Laura Johnson, Nicholas Morgan, Suzet Tanya Lereya, Julian Edbrooke-Childs

**Affiliations:** 1 Evidence Based Practice Unit Anna Freud National Centre for Children and Families London United Kingdom

**Keywords:** mHealth, mental health, peer support, COVID-19, well-being, young people

## Abstract

**Background:**

Digital peer support is an increasingly used form of mental health support for young people. However, there is a need for more research on the impact of digital peer support and why it has an impact.

**Objective:**

The aim of this research is to examine young people’s experiences of using a digital peer support tool: MeeToo. After the time of writing, MeeToo has changed their name to Tellmi. MeeToo is an anonymous, fully moderated peer support tool for young people aged 11-25 years. There were two research questions: (1) What impacts did using MeeToo have on young people? (2) Why did using MeeToo have these impacts on young people?

**Methods:**

A mixed methods study was conducted. It involved secondary analysis of routinely collected feedback questionnaires, which were completed at two time points (T1 and T2) 2-3 months apart. Questionnaires asked about young people’s (N=876) experience of using MeeToo, mental health empowerment, and well-being. Primary data were collected from semistructured interviews with 10 young people.

**Results:**

Overall, 398 (45.4%) of 876 young people completed the T1 questionnaire, 559 (63.8%) completed the T2 questionnaire, and 81 (9.2%) completed both. Descriptive statistics from the cross-sectional analysis of the questionnaires identified a range of positive impacts of using MeeToo, which included making it easier to talk about difficult things, being part of a supportive community, providing new ways to help oneself, feeling better, and feeling less alone. Subgroup analysis (paired-sample *t* test) of 58 young females who had completed both T1 and T2 questionnaires showed a small but statistically significant increase in levels of patient activation, one of the subscales of the mental health empowerment scale: time 1 mean=1.83 (95% CI 1.72-1.95), time 2 mean=2.00 (95% CI 1.89-2.11), *t*_59_=2.15, and *P*=.04. Anonymity and the MeeToo sense of community were identified from interviews as possible reasons for why using MeeToo had these impacts. Anonymity helped to create a safe space in which users could express their feelings, thoughts, and experiences freely without the fear of being judged by others. The MeeToo sense of community was described as a valuable form of social connectedness, which in turn had a positive impact on young people’s mental health and made them feel less isolated and alone.

**Conclusions:**

The findings of this research showed a range of positive impacts and possible processes for young people using MeeToo. Future research is needed to examine how these impacts and processes can be sustained.

## Introduction

Evidence suggests that peer support may improve mental health, social functioning, and quality of life [1], particularly during the COVID-19 pandemic [2]. For example, research indicates the effectiveness of using digital resources in peer support work to help young people experiencing psychosis [3]. There is also research illustrating how digital peer support can help people with severe mental health difficulties in their recovery process by encouraging a culture of health and ability [4]. Digital peer support has the potential to reach more young people in need of support than young people being able to access face-to-face peer and other forms of support [5]. This is particularly the case during the periods of quarantine and lockdown due to the COVID-19 pandemic, where digital peer support can be more readily available and accessible to young people [2]. Indeed, research suggests that digital mental health interventions are effective in mitigating psychosocial consequences of social distancing, quarantine, and other restrictions due to the COVID-19 pandemic [6].

In this regard previous studies indicate that the desirable aspects of digital mental health peer support systems are matching peers according to shared interests and identities that they self-identify with, not matching peers according to their mental health diagnosis, and highlighting through social media or other online mediums that discussing mental health is safe in a peer support community [7]. Other important aspects that emerged from previous research on digital peer support platforms were: to educate peers on how to offer support without suggesting unhelpful coping strategies, to guarantee some anonymity and control over how peers present themselves to each other on these platforms, and to provide adequate information to potential peers to facilitate their decision on whether they would like to start befriending their matched peer prior to connecting with them [7].

In addition, Kenny et al’s research [8] indicates that young people identified 6 main factors when asked about the development of a mental health app prototype: safety and engagement, functionality and social interaction, awareness, accessibility, gender, and young people in control as important factors. Regarding safety and engagement, the app must be safe in terms of confidentiality, cyberbullying, and stigma and it also must be engaging and user-friendly. Concerning functionality and social interaction, the app must have the useful and relevant function of providing mental health support, as well as allowing young people to interact with one another in an anonymous way. With respect to awareness and accessibility, the app should also be promoted online and offline to raise awareness of mental health among young people and must be easily accessible. Young people also highlighted that there are gender differences among users on the extent to which they would engage with the app. Finally, young people expressed the importance of being in control of how and the extent to which they use the app.

There is also research on digital peer support programs suggesting that these types of interventions encourage young people to self-refer to mental health services and seek help among their social networks, thus effectively combatting mental health stigma among young people [9]. In this respect, evidence suggests that there are 3 kinds of connectedness that are essential for the development of effective digital mental health and well-being tools: professional, self, and peer [10]. Professional connectedness refers to young people’s trust in the credibility and authenticity of the tool, similar to the requirements for face-to-face mental health and well-being support. Self-connectedness refers to young people feeling they can share their own experiences appropriately and developing new insights and support strategies. Finally, peer connectedness refers to young people being able to connect with other young people with similar experiences in a safe but meaningful way, often one of the most challenging aspects of digital mental health and well-being support tools.

However, a systematic review suggests a lack of research in digital peer support as it is often used in a supplementary way to face-to-face interventions, and thus the individual effectiveness of digital peer support is not accurately investigated [2]. In addition, research suggests that although there is some evidence of the effectiveness of digital peer support, such as mental health apps, studies remain imprecise on how effective these apps are compared to standard mental health care [11].

MeeToo is a fully moderated anonymous digital peer support tool (app) that is widely used by young people in a range of settings [12]. After the time of writing, MeeToo has changed their name to Tellmi. It is freely available for young people aged 11-25 years [13]. A user is able to see moderated posts from other users who are aged +/–2 years of the user’s age, except for users 18-25 years old who are able to see moderated posts from users 18-25 years old. Trained and paid moderators review all posts 24/7 to assess risks and tag posts by theme. Other users can post replies to a post, and these replies are also moderated. Trained “super peers” review posts and make sure no one is left without a response. Super peers are university students from psychology, medicine, and other mental health relevant departments who have completed the structured and monitored MeeToo Super Peer Programme, which is acceptable to be integrated into a degree course as a placement option. The program is offered remotely as super peers do not have to be based in the United Kingdom. In addition, counsellors are available if there are any serious or safeguarding concerns. Posts can be filtered by topic, and there is also a library of mental health resources. Users generally find out about MeeToo through peers, schools, or mental health support services. Apart from research collaboration, the authors of this paper have no other professional or personal involvement in MeeToo.

Indeed, there is a need for more evidence of the impact of digital peer support and how it might achieve this impact. This question is particularly important now because young people face a period of increased social isolation and interruption to regular peer activities due to social distancing [14,15]. In this respect, according to Newlove-Delgado et al [16], examining the mental health of children and young people during the COVID-19 pandemic, 39.2% of 6- to 16-year-olds experienced a deterioration in their mental health since 2017 and 21.8% experienced an improvement. In addition, among 17- to 23-year-olds, 52.5% experienced a worsening of their mental health and 15.2% experienced progress. This worsening of mental health was greater for young females than for young males, and levels of mental health difficulties were already higher for young females before the COVID-19 pandemic. A recent study examined one-year follow-up data for two groups of young people, one in 2018 (before COVID-19) and one in 2019 (during COVID-19) [17]. They found that young people in the 2019 group had higher levels of depressive symptoms and lower levels of satisfaction, with a greater negative impact on young females than on young males. They estimate that had COVID-19 not occurred, 6% fewer young people would have experienced high levels of depressive symptoms.

In this regard, young people experienced disruptions in their learning because of the closing of schools and the restricted face-to-face interaction with their peers [14]. Indeed, COVID-19 regulations have negatively impacted young people’s mental health and well-being, as studies illustrate how many young people experienced COVID-19–related fear as well as depressive and anxious symptoms [15]. These mental health difficulties were prevalent in older adolescents, females, and young people with neurodiversities or chronic physical conditions [15]. It is important that we better understand how to support young people to use digital mental health self-care, given the current global pandemic and corresponding increased stress and adversity.

The aim of this research is to examine young people’s experiences of using a digital peer support tool, MeeToo. There were two research questions:

- What impacts did using MeeToo have on young people?
- Why did using MeeToo have these impacts on young people?

## Methods

### Rationale

A concurrent triangulation design mixed methods study [18] was performed to address the two research questions. During setup, a Logic Model was coproduced by researchers at the Anna Freud Centre and the MeeToo team (see Multimedia Appendix 1) [19] to provide a transparent description of the conceptualization of MeeToo and to identify data collection needs. Quantitative and qualitative data were collected separately, with the quantitative data used to address research question 1 on the impacts of using MeeToo on young people and the qualitative data used to address the related research question 2 on why using MeeToo has these impacts.

### Quantitative Methodology

#### Data Collection

Secondary analysis of anonymized routinely collected feedback questionnaire data was conducted. The questionnaire data were collected over a period of 5 months from January to May 2021. These questionnaires were collected at time point 1 (T1), from January to February, and approximately 2-3 months later at time point 2 (T2), from April to May. As these were routinely collected data, respondents had been using MeeToo for varying amounts of time when they completed the questionnaires (see Table 1). The anonymized data were securely transferred to the research team. The questionnaires asked 7 bespoke questions about the impact of using MeeToo (eg, “Using MeeToo makes it easier for me to talk about difficult things”), with a 6-point response option from “strongly agree” to “strongly disagree” and an additional “I don’t know” option. The 17-item Mental Health Empowerment Scale that the authors previously developed asked about young people’s levels of mental health patient activation (7 items, eg, “I know how to look after my mental health”), levels of availability of social support (3 items, eg, “I have friends I can talk to when I feel bad”), their access to information and support for their mental health (4 items, eg, “I can find information that I trust if I have questions about my mental health”), and their confidence in said information (3 items, eg, “I will be listened to when getting help about my mental health”). The responses were rated on a 3-point scale from “agree” to “disagree,” with higher scores indicating higher levels of mental health empowerment. Finally, well-being was measured using the 4-item Outcome Rating Scale (ORS) [20]. The ORS assessed young people’s personal, relational, social, and general well-being (eg, “How are you doing overall?”), and it was rated on 10 cm visual analog scales from “not good” to “doing great.” Responses were then scored on a 0-10 scale by centimeter, with higher scores indicating high levels of well-being, and total average scores were then computed. Internal consistency (Cronbach *α*) for subscales of the Mental Health Empowerment Scale and overall well-being are shown in Table 1.

**Table 1:** MeeToo usage statistics (how long participants reported they had been using MeeToo).^a^

Period of using MeeToo	T^b^1 participants (n=398), n (%)	T2 participants (n=559), n (%)
Just started	199 (50)	175 (31)
<1 week	32 (8)	35 (6)
2-4 weeks	20 (5)	45 (8)
>1 month	147 (37)	304 (54)

^a^Percentages may not add up to 100% due to rounding.

^b^T: time point.

#### Analysis

Quantitative data were analyzed using the STATA 16 [21]. The survey data were collected by the MeeToo app at two time points, at T1 and 2-3 months later at T2. Results were analyzed using a cross-sectional sample of those who completed one survey and a longitudinal sample of those who completed both surveys. Analysis of the cross-sectional sample involved descriptive statistics. Analysis of the longitudinal sample involved paired-sample *t* tests to explore changes over time in the four subscales of the Mental Health Empowerment Scale and the ORS. Given the gender imbalance in the data, these analyses were performed for females and males separately as well as together.

### Qualitative Methodology

#### Participants

A qualitative methodology was used involving individual semistructured interviews with 10 young users from four different schools, of which 6 (60%) users identified as female and 4 (40%) as male, and the young people were aged between 14 and 18 years. The sample size enabled us to hear variations in experiences and then close recruitment when there was sufficient consistency in the young people’s responses [22]. Participants were recruited from 6 schools in the United Kingdom that used the MeeToo app. In addition, participants came across the opportunity through the app, schools, social media, the Anna Freud Centre website, and the MeeToo website. Participants could express interest by completing a Microsoft Teams online contact form or by directly emailing the research team. The research team then contacted the participants to answer any questions they might have had about the study and their involvement. At this stage, the research team also shared with the participants the information sheet and the Microsoft Teams online consent form.

#### Ethical Considerations

After the participants’ queries were addressed, they provided informed consent by completing the online consent form. Ethical approval was received from the University College London Research Ethics Committee (Ref. 14037/004). Informed consent or assent to take part in the evaluation was obtained by all participants online as was parental consent for all young people under the age of 16 years. The participants were not reimbursed for taking part in the qualitative interviews.

#### Procedure

The interviews were jointly conducted by the Research Assistant and a Peer Researcher to have a young person’s perspective on the research process in order for it to be as inclusive and as representative as possible. Indeed, mental health and health research has highlighted the importance of involving members of the public in research processes in order to be as inclusive and representative as possible [23,24]. The peer researcher is a paid young person, aged 15-25 years, with either direct or indirect experience of mental health difficulties. Indirect experience includes, for example, a young person with a parent or carer with experience of mental health difficulties. Peer Researchers have also an interest or experience in mental health research. Indeed, the Peer Researcher supported us on this project, with the design of the study, data collection, analysis, and write-up. In particular, the Peer Researcher supported us in designing the topic guide for the interviews, which was developed following the Logic Model (see Multimedia Appendix 1) [19]. The topic guide included questions about when and how participants used the app, their experience using the app, whether they experienced any changes after using the app, and any recommendations for improvement. The Peer Researcher also assisted in conducting the interviews as well as providing feedback and input on the interpretation of the coding of the interviews and on identification of themes in the analysis. Interviews lasted from 30 minutes up to an hour and were conducted online on either Microsoft Teams calls or video calls over a period of 3 months between March and May 2021. In addition, interviews were conducted either after participants had used the MeeToo app or while they were still accessing and using the app.

#### Analysis

All interviews were audio-recorded using an encrypted Dictaphone and transcribed verbatim by an independent transcription service. The transcripts were then reviewed by the research team before being analyzed. In addition, all interviews were anonymized at the point of transcription (eg, names of people and places given by the participants in their interviews). The interview transcriptions were analyzed and coded using thematic analysis through NVivo (QSR International), a qualitative research analysis software program [25]. Thematic analysis was chosen as it can offer insight into people’s views and experiences of a certain topic [26], such as digital peer support platforms [27,28].

## Results

We first describe the study sample and then present the MeeToo usage data for context. We then present the main results by each research question.

### Participants

Overall, 876 young people completed at least one questionnaire. Of these, 317 (36.2%) young people completed the questionnaire at T1 only, 478 (54.6%) completed it at T2 only, and 81 (9.2%) completed it at both T1 and T2. As the matched T1 and T2 questionnaire data were relatively underpowered to detect significant differences, we mainly focused on the young people’s responses at T1 (n=398, 45.4%) and at T2 (n=559, 63.8%) separately. Demographic characteristics are shown in Table 2. Across both surveys, almost half of the young people were aged 13-15 years and a third were aged 16-18 years. Smaller numbers of responses were received from 11-12-year-olds or those aged 19 years or older. The majority of respondents were young females (T1 n=256, 64%; T2 n=423, 76%). We used the terms “young females” and “young males” inclusively, reflecting gender self-identity.

**Table 2:** Demographic characteristics.^a^

Characteristics		T^b^1 participants (n=398), n (%)		T2 participants (n=559), n (%)
**Age (years)**				
	11-12	48 (12)	37 (7)	
	13-15	187 (47)	260 (47)	
	16-18	133 (33)	198 (35)	
	≥19	30 (8)	64 (11)	
**Gender^c^**				
	Young females	256 (64)	423 (76)	
	Young males	104 (26)	71 (13)	
	Not reported or missing	38 (10)	65 (12)	

^a^Percentages may not add up to 100% due to rounding.

^b^T: time point.

^c^The three-response options for gender were “female,” “male,” and “prefer not to say.”

### Usage of MeeToo

In this section we discuss some questionnaire and qualitative data on the usage of MeeToo. Usage, acceptability, and engagement were not central to the research questions, and therefore, these data are presented to contextualize the key findings.

Across both questionnaires, most young people had either only just started using MeeToo or had been using it for some time (at least a month). At T1, half of young people (n=199, 50%) had just started and over a third (n=147, 37%) had been using it for at least a month. At T2, the pattern was reversed: over half of young people (n=304, 54%) had been using it for at least a month and less than a third (n=175, 31%) had just started. This possibly suggests greater levels of engagement with MeeToo at the end of the evaluation period.

The qualitative data suggested that young people found the MeeToo app easy to use.

I found it very easy to use. It’s very simple. It’s just simply when you feel something that you need to post, I just go on there and I can feel comfortable enough to post it. It’s very simple steps, and I don’t think the checking process takes very long either. It takes about 10, 15 minutes and then it’s on the app automatically. Yeah, it was very easy to use as well and the support programs on the side, they’re very easy to access as well, which I really like.

I found it very simple to get around really, which was very good. And like everything was labeled in a good way, and you could search for keywords.

This encouraged young people to use the app more.

The easiness of the app really encouraged me to pick it up more often. It’s more like an automatic response when I’m feeling overwhelmed, I feel very comfortable that I can just immediately pick up my phone and go onto the MeeToo app. Yeah, it’s almost like a reflex now for me.

A few challenges with using MeeToo were reported by young people. Some young people described how an improved ability to filter content, especially on the home page of the app, would enhance usage by making it easier for young people to avoid specific topics that were sensitive to them. Some young people described inadvertently viewing such topics as deterring them from using the app and as resurfacing distressing thoughts and feelings. One young person suggested that there could be a feature on the home page providing users with the option to block sensitive content in order for the users to positively engage with the app. MeeToo is improving the ability to filter content, especially on the home page of the app, but this was not available when young people took part in this study.

There were lots of details and words which I would have just felt very uncomfortable to read, if I was back in the situation I was in. And I think just seeing that kind of just put a bad image of the app in my mind. It’s such a good idea, and I think it’s so good.

It's just there are really just two topics that I really just don't want to read about, more just as a way of…I don't like even always thinking about it.

### What Impacts Did MeeToo Have on Young People?

Descriptive statistics for the bespoke questionnaire data are shown in Table 3. As expected during COVID-19, young people reported low levels of well-being at T1 and T2 in the questionnaire data. Nevertheless, across T1 and T2, the majority of young people (259/398, 65%, to 469/559, 84%) agreed that using MeeToo:

- Made it easier to talk about difficult things
- Connected them to people with similar problems
- Enabled them to feel useful by helping others
- Was a supportive community
- Provided new ways to help oneself
- Helped them feel better
- Helped them feel less alone

**Table 3:** Descriptive statistics for bespoke questionnaire items.^a,b^

Questionnaire items		T^c^1 participants (n=398), n (%)	T2 participants (n=559), n (%)
**Easier to talk about difficult things**			
	Agree	272 (68)	434 (78)
	Disagree	21 (5)	31 (6)
	Missing	105 (26)	94 (17)
**Connects to people with similar problems**			
	Agree	309 (78)	456 (82)
	Disagree	22 (6)	30 (5)
	Missing	67 (17)	73 (13)
**I feel useful helping others**			
	Agree	301 (76)	440 (79)
	Disagree	16 (4)	30 (5)
	Missing	81 (20)	89 (16)
**MeeToo is a supportive community**			
	Agree	318 (80)	469 (84)
	Disagree	10 (3)	13 (2)
	Missing	70 (18)	77 (14)
**New ways to help myself**			
	Agree	259 (65)	397 (71)
	Disagree	34 (9)	50 (9)
	Missing	105 (26)	112 (20)
**I feel better when I use MeeToo**			
	Agree	262 (66)	404 (72)
	Disagree	31 (8)	45 (8)
	Missing	105 26()	110 (20)
**I feel less alone with MeeToo**			
	Agree	284 (71)	439 (79)
	Disagree	26 (7)	34 (6)
	Missing	88 (22)	86 (15)

^a^Questionnaire items are paraphrased in the table to facilitate interpretation.

^b^Percentages may not add up to 100% due to rounding.

^c^T: time point.

Descriptive statistics for the scales used in the questionnaire are shown in Table 4. For the 81 young people who completed both T1 and T2 questionnaires, there were no significant differences in well-being in the group and gender subgroup analyses. However, in the subgroup analysis examining young females, overall well-being increased by 0.83 points, from 3.34/10 (95% CI 2.76-3.93) at T1 to 4.17/10 (95% CI 3.54-4.81) at T2, although the difference was not statistically significant: *t*_57_=1.97, *P*=.05. Similarly, when looking at change over time in raw patient activation scores, there were no significant differences in the group and subgroup analyses examining young males. However, in the subgroup analysis examining young females, levels of patient activation increased from a mean of 1.83/3 (95% CI 1.72-1.95) to 2.00/3 (95% CI 1.89-2.11), a small but statistically significant increase: *t*_59_=2.15, *P*=.04.

We compared age, gender, duration of use, and overall well-being for young people with complete T1 and T2 questionnaires and with only the T1 or T2 questionnaire complete only on baseline characteristics (ie, scores at T1 for those with complete T1 and T2 questionnaires or only the T1 questionnaire and scores at T2 for those with only the T2 questionnaire). There were no significant differences in the distributions of females and males (*χ*^2^_1_=0.944, *P*=.33) or age categories (*χ*^2^_3_=6.94, *P*=.07). Young people with complete T1 and T2 questionnaires had higher levels of self-reported MeeToo usage than young people with only the T1 or T2 questionnaire complete (*χ*^2^_3_=48.24, *P*<.001). In particular, there were more young people with T1 and T2 questionnaires complete who had been using MeeToo for a month or more (62/81, 77%) than those with only the T1 or T2 questionnaire complete (308/795, 39%). In addition, there were fewer young people with complete T1 and T2 questionnaires who had only just started using MeeToo (8/81, 10%) than those with only the T1 or T2 questionnaire complete (366/795, 46%). Young people with complete T1 and T2 questionnaires had lower levels of well-being than young people with only the T1 or T2 questionnaire complete: *t*_830_=2.81, *P*=.01; complete T1 and T2 questionnaire mean=4.01(95% CI 3.55-4.47, n=80); complete T1 or T2 questionnaire mean=4.81 (95% CI 4.63-4.98, n=752). These findings suggest that longitudinal analyses for young people with complete T1 and T2 questionnaires are less likely to generalize to young people who have been using MeeToo for shorter periods or with higher levels of well-being.

**Table 4:** Internal consistencies and descriptive statistics for questionnaire scales.

Scale	T^a^1				T2			
	N	Cronbach *α*	Mean (SD)	N		Cronbach *α*	Mean (SD)	
Overall well-being	382	.91	5.07 (2.58)	529		.85	4.44 (2.23)	
Patient activation	388	.87	2.11 (0.54)	536		.85	1.97 (0.5)	
Social support	388	.73	2.05 (0.68)	536		.67	1.82 (0.63)	
Help	388	.79	2.16 (0.62)	536		.76	2.02 (0.6)	
Confidence in help	388	.84	2.23 (0.66)	536		.72	2.09 (0.65)	

^a^T: time point.

### Why Did MeeToo Have These Impacts on Young People?

Two themes were identified in response to this research question: (1) “I don't like talking to people I know, but having an anonymous platform […] is a really good thing” and (2) the MeeToo sense of community. In the interviews, young people described how the anonymity of MeeToo enabled them to authentically share experiences and support other young people as it was a safe space. Connecting with other young people with similar experiences fostered a sense of community, and belonging to this community helped young people feel less alone and feel better.

#### Theme 1: I Don’t Like Talking to People I Know, but Having an Anonymous Platform […] Is a Really Good Thing

Young people reported in the interviews that anonymity was a central reason why using MeeToo had the aforementioned impacts. Anonymity facilitated their ability to voice their feelings, thoughts, and experiences freely without fear of being judged by others, creating a safe space.

I think it's a really good app to use, because sometimes you can't talk to your friends or family about it. And all our usernames are anonymous, which is nice, so you don't know, like, for instance, someone could be my best friend, or whatever, but I don't know that, and they don't know that it's me. I like the anonymous aspect of it.

All quotes from young people

Anonymity facilitated this process for young people, particularly if they did not feel comfortable opening up about their difficulties with people they know.

I know for me, especially, I don't like talking to people I know, but having an anonymous platform to find people that feel the same way or can give you advice on anything is a really good thing.

By enabling young people to talk openly, they were able to connect with others who had similar experiences. This then encouraged them to provide support to others in a genuine way, particularly in times of stress during lockdown and school assessment periods. Anonymity also mitigated any concerns about worrying other people by talking about one’s own challenges.

I thought it was cool that you get to post anonymously and get to see. My main thing that I really liked about the app is that a lot of people have similar opinions to you. And when I went on it, I saw something someone said and that resonated with me, because I felt the same way. So, I replied, and we had this chat, and it was helpful for me. And I hope that it was helpful for that person as well. But I really just like the fact that it's anonymous and you get to help each other.

I think a lot of people struggle with not wanting to worry others with their own problems. So I think helping people via the app is a very effective way to do so.

Young people described anonymity as particularly important because of mental health stigma, even causing some to hide that they have the MeeToo app on their phone.

I know a lot of people try and hide it. I don't know how people can improve on that, but I just know it's a common thing, where people don't want to talk about mental health, as such, and they literally hide the app on their phone, and hide the notifications.

The anonymity of interaction in MeeToo enabled young people to connect with one another in a more authentic way, as they were able to be more open.

So, it's definitely helped during the app, because it's a good way for people to come and talk to other humans without actually having to go up to the person and not being identified. So, I think it's definitely meant that quite a lot of the stuff on there is very genuine and it's quite good to see that people are making an impact on other people's lives.

Young people described anonymity as making it easier for them to support others with similar experiences, which in turn built their own confidence.

When my family was going through some stuff at the start of lockdown, it was quite nice to scroll down through the family section—I searched it up on the search bar—and it was really nice to scroll down and see that I could talk about how I’d dealt with it with other people, that there were coping mechanisms and breathing techniques that I used to get through those hard times myself. It was nice to share them and see if they actually helped people.

#### Theme 2: The MeeToo Sense of Community

The safe space created by the anonymity of MeeToo helped create a sense of community, where young people with similar experiences were sharing with and supporting one another. This sense of community fostered a strong sense of connection, leading to the aforementioned impacts of using MeeToo. Young people explained how the app fostered a sense of community that helped them relate to and connect with their peers experiencing mental health difficulties, particularly during lockdown.

I think at the time, because it was in the middle of lockdown, I was definitely struggling with my mental health quite a lot around that time. I tried something else before which didn’t really work out, a different application. But MeeToo was definitely a big change because there was more of a community, it was more people sharing their experiences, and I really liked that about it. It was all these people and I actually related with some of the stuff they said.

I search for stuff that I think, if I find a comment that maybe I relate to, I can help someone. You feel very in touch with people on that app, there’s definitely some kind of not direct friendship but you feel very connected to people if you’re experiencing the same thing.

Anonymity and the safeguarding in MeeToo encouraged a sense of a connected and also safe community.

I felt much more comfortable on there than I did with many other apps because it felt very much like a community. I don’t want to compare it to [social media], but it was like that but with a lot more safeguarding around it, which I really like because it felt very safe, it felt very secure.

Connecting through the MeeToo community was described as a valuable source of social connectedness, which in turn had a positive impact on young people’s mental health. It also helped people feel less alone and isolated as other people had similar challenging experiences, empowering young people to talk about mental health more in the app and offline.

It’s really nice to feel like you can help someone. I’ve always loved helping people, just telling people how much they mean to me. It makes me happy seeing other people happy. To feel like I’ve actually helped someone, that really boosts my mood.

And I thought anxiety was like this really ultra, awful mental illness, which happens to very few people, and just reading the repetitiveness of posts around anxiety, I just realized there are so many people must have it or suffer with it.

So I guess when I'm talking to my mates or talking to people who are looking for support, I can tell them quite comfortably, you know: you're not the only one, this happens to quite a few people. So you can relate to it more.

## Discussion

### Principal Findings

The aim of this research was to examine young people’s experiences of using a digital peer support tool, MeeToo. In so doing, we addressed two research questions: (1) What impacts did using MeeToo have on young people? (2) Why did using MeeToo have these impacts on young people? A mixed methods study was conducted, which involved secondary analysis of routinely collected questionnaire data and interviews with young people.

A range of positive impacts of using MeeToo were reported by young people in the questionnaires, which included making it easier to talk about difficult things, being part of a supportive community, providing new ways to help oneself, feeling better, and feeling less alone. A smaller number of young people completed questionnaires at both T1 and T2. Here, subgroup analysis showed that young females had a significant increase in patient activation over time, suggesting that they felt more knowledgeable and confident to manage their mental health. Nevertheless, without a randomized controlled design, inferences about causation cannot be made. Subgroup analysis was performed by gender as the majority of survey respondents were young females. This is in line with previous research showing that more young females than young males engage with digital support tools and in research on their experiences of these tools [7,9]. It is also not surprising, as previous research has shown higher levels of mental health difficulties in young females than in young males and a greater negative mental health impact of the COVID-19 pandemic on young females [17]. In addition, a few challenges with using MeeToo were reported by young people, such as inadvertently viewing sensitive content on the app. Regarding this, young people suggested that there could be a feature on the home page of MeeToo that could offer users the option to block or filter sensitive content so that they can engage more positively with the app. In this respect, the MeeToo team is now improving the ability of the app to filter content.

Two themes were identified from the interviews about why using MeeToo had these impacts on young people. The first theme was “I don't like talking to people I know, but having an anonymous platform […] is a really good thing.” Young people describe anonymity as a central reason why using MeeToo had the aforementioned impacts. Anonymity helped create a safe space in which users could express their feelings, thoughts, and experiences freely without the fear of being judged by others, particularly when they were not comfortable sharing difficulties with people they know. This reflects previous research on digital peer support platforms suggesting that anonymity fosters a sense of separation from pre-existing ties, facilitating users to open up about difficulties [7]. This finding is also in line with previous research identifying self-connectedness (reflecting on one’s own feelings, thoughts, and experiences) as important for engagement in digital mental health support [10]. Anonymity enabled young people to connect with others who had similar experiences, as interactions were more authentic. It also helped young people feel confident and empowered to provide support in a genuine way to others, both online and offline, especially about difficulties they had experienced during the COVID-19 pandemic. This relates to previous research highlighting the importance of educating peers on how to offer support online to foster a sense of empowerment among young people to share their difficulties and help one another [7].

The second theme was the MeeToo sense of community, which was created by anonymity and the ability to authentically connect with others. This sense of community helped young people relate to and connect with peers, which was particularly valued during lockdowns. The MeeToo sense of community was described as a valuable form of social connectedness, which in turn had a positive impact on young people’s mental health and made them feel less isolated and alone. These findings are in line with research on digital peer support platforms indicating that stigma regarding mental health difficulties within peer support spaces decreases when users share similar experiences and interests [7,9]. This theme is also in line with previous research identifying peer connectedness as important for engagement in digital mental health support [10]. The safeguarding in MeeToo made young people feel like the community was safe. Being supported by others with similar experiences appeared to important for establishing the credibility of MeeToo, as support was provided by experts by experience. Again, these findings are in line with previous research identifying professional connectedness as important for engagement, which in digital support refers to trust in the safety and authenticity of the tool [10].

### Limitations

Limitations of this research include the small number of young people with paired T1 and T2 questionnaire data in the secondary analysis, which meant the longitudinal analyses were likely underpowered. The use of routine data and the lack of controlled conditions likely resulted in the small amount of longitudinal data. Still, we do not have data on whether young people who completed the T1 questionnaire but not the T2 questionnaire stopped using MeeToo or continued but did not complete the T2 questionnaire. These findings suggest that longitudinal analyses for young people with complete T1 and T2 questionnaires are less likely to generalize to young people who have been using MeeToo for shorter periods or with higher levels of well-being. As previously mentioned, without a randomized controlled design, inferences about causation cannot be made. Other limitations of routinely collected data also apply to this research [29]. Self-reported usage data were collected from questionnaires and interviews to contextualize the findings. There was not a greater focus, as it was not central to the research questions and MeeToo is already widely used [12]. Nevertheless, future research on MeeToo usage, acceptability, and engagement is recommended, given the challenge of sustained engagement with digital mental health support tools [10]. For example, a randomized controlled design could be used to examine the effectiveness of different engagement strategies in MeeToo, such as regular personalized encouragement messages from super peers. Similarly, future research should also examine the experiences of MeeToo, with a focus on young people from marginalized groups to examine the extent to which it is inclusive of their needs. Indeed, the majority of young people who completed the questionnaires were young females, suggesting that future research with young people with different gender identities is needed. In particular, we cannot say whether young people with different gender identities did not complete the questionnaires, because they were less likely to use MeeToo or because the questionnaires were less inclusive. Regarding the interviews, although the sample size enabled us to hear variations in experiences and then close recruitment when there was sufficient consistency in young people’s responses, the sample size was still relatively small. Future research could recruit larger qualitative samples, for example, by offering a range of ways for participants to take part (eg, interviews, free-text questionnaires, photoelicitation). Qualitative research with young people who stopped using MeeToo would be particularly useful to examine the question of sustained engagement.

### Conclusion

The aim of this research was to examine young people’s experiences of using a digital peer support tool, MeeToo. A range of positive impacts of using MeeToo were reported by young people in questionnaires, which included making it easier to talk about difficult things, being part of a supportive community, providing new ways to help oneself, feeling better, and feeling less alone. Anonymity and the MeeToo sense of community were identified from interviews as possible reasons for why using MeeToo had these impacts. Anonymity helped create a safe space in which users could express their feelings, thoughts, and experiences freely without the fear of being judged by others. The MeeToo sense of community was described as a valuable form of social connectedness, which in turn had a positive impact on young people’s mental health and made them feel less isolated and alone. Future research is needed to examine how these impacts and processes can be sustained.

## Appendix

Multimedia Appendix 1 MeeToo app Logic Model.

## Abbreviations

ORS: Outcome Rating Scale
