# Supplementary figures and images for: Experiences of Using the Digital Support Tool MeeToo: Mixed Methods Study
Source: JMIR Pediatr Parent. 2022 Oct 20;5(4):e37424. doi: 10.2196/37424 (PMC9629342; doi:10.2196/37424)

**Appendix 1**


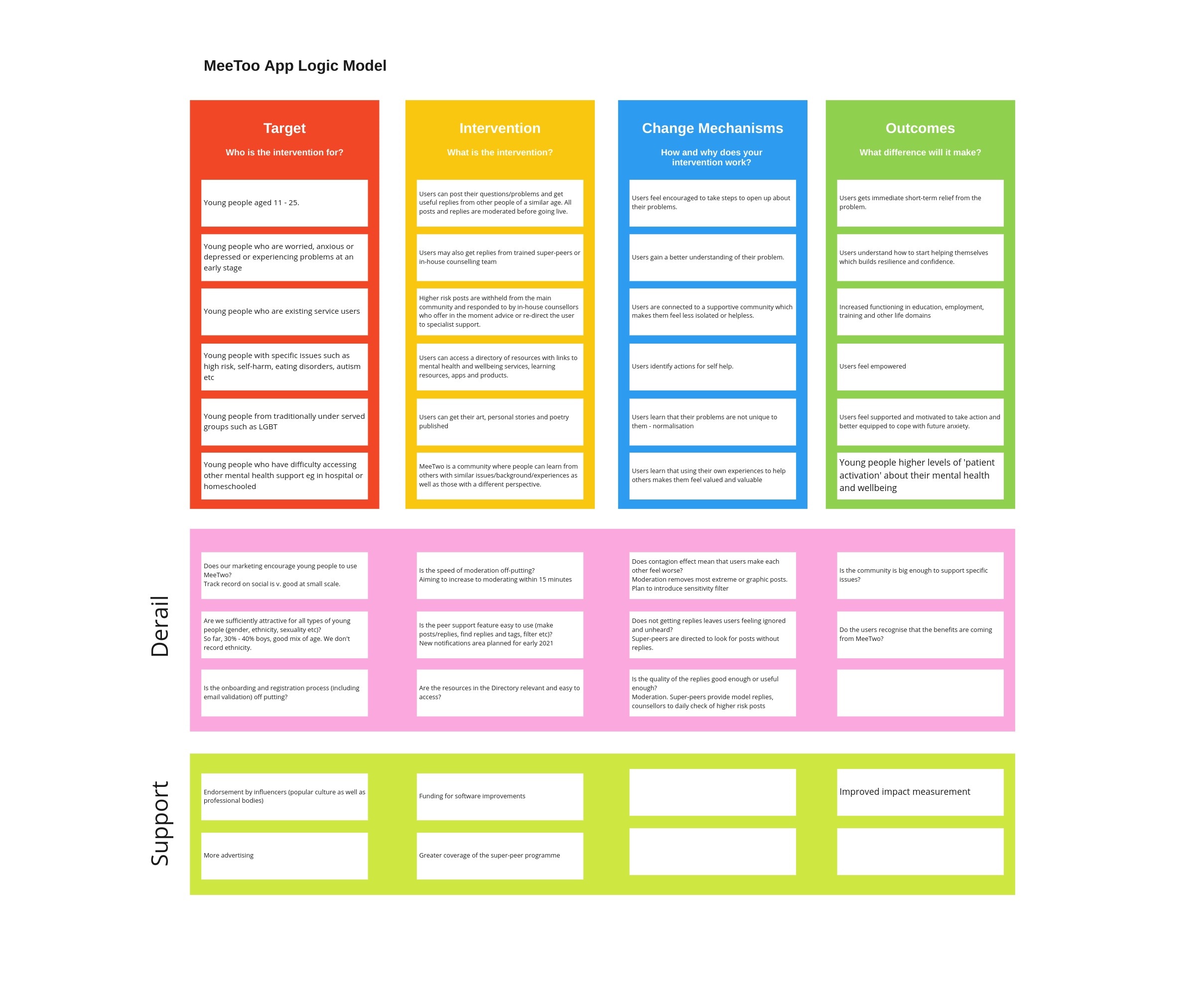

Supplement: Multimedia Appendix 1 [file pediatrics_v5i4e37424_app1.docx]
